# Neuropathic Pain Dysregulates Gene Expression of the Forebrain Opioid and Dopamine Systems

**DOI:** 10.1007/s12640-020-00166-4

**Published:** 2020-02-05

**Authors:** Agnieszka Wawrzczak-Bargieła, Barbara Ziółkowska, Anna Piotrowska, Joanna Starnowska-Sokół, Ewelina Rojewska, Joanna Mika, Barbara Przewłocka, Ryszard Przewłocki

**Affiliations:** 1 Department of Molecular Neuropharmacology, Maj Institute of Pharmacology, Polish Academy of Sciences, 12 Smętna Street, 31-343 Kraków, Poland; 2 Department of Pain Pharmacology, Maj Institute of Pharmacology, Polish Academy of Sciences, 12 Smętna Street, 31-343 Kraków, Poland

**Keywords:** Chronic pain, Neuropathy, Mesostriatal system, Dopamine, Opioids, Nucleus accumbens

## Abstract

Disturbances in the function of the mesostriatal dopamine system may contribute to the development and maintenance of chronic pain, including its sensory and emotional/cognitive aspects. In the present study, we assessed the influence of chronic constriction injury (CCI) of the sciatic nerve on the expression of genes coding for dopamine and opioid receptors as well as opioid propeptides in the mouse mesostriatal system, particularly in the nucleus accumbens. We demonstrated bilateral increases in mRNA levels of the dopamine D1 and D2 receptors (the latter accompanied by elevated protein level), opioid propeptides proenkephalin and prodynorphin, as well as delta and kappa (but not mu) opioid receptors in the nucleus accumbens at 7 to 14 days after CCI. These results show that CCI-induced neuropathic pain is accompanied by a major transcriptional dysregulation of molecules involved in dopaminergic and opioidergic signaling in the striatum/nucleus accumbens. Possible functional consequences of these changes include opposite effects of upregulated enkephalin/delta opioid receptor signaling vs. dynorphin/kappa opioid receptor signaling, with the former most likely having an analgesic effect and the latter exacerbating pain and contributing to pain-related negative emotional states.

## Introduction

Chronic pain is a common medical problem affecting nearly 20% of adults in Europe and North America (Breivik et al. 2006; Nahin 2015). Despite major progress in elucidating the mechanisms of nociception (Basbaum et al. 2009), many cases of chronic pain remain resistant to medical treatment, particularly those of the neuropathic type of pain, i.e., resulting from lesions or diseases of the somatosensory nervous system (Turk 2001; Dickenson and Suzuki 2005; IASP Terminology 2017).

Chronic pain develops most often from acute pain states elicited by tissue or nerve damage and is most likely underlain by maladaptive plastic changes in the central nervous system produced in response to prolonged nociceptive stimulation. Whereas a large number of pain-related molecular alterations have been described in the spinal cord (Basbaum et al. 2009; Kuner 2010; Mika et al. 2013; Rojewska et al. 2014, 2018), there is also an emerging recognition of a vital role of supraspinal centers in the development and maintenance of chronic pain conditions. This applies not only to the classical components of the somatosensory pathway but also to limbic forebrain regions, which may contribute to the affective and cognitive aspects of pain perception, and exert descending control on nociception (Tracey and Mantyh 2007; Bushnell et al. 2013).

Among them, attention has focused on the mesostriatal dopamine system encompassing projections from the substantia nigra and ventral tegmental area to the dorsal and ventral striatum (the latter consisting chiefly of the nucleus accumbens, NAc) (Wood 2006; Baliki and Apkarian 2015; Taylor et al. 2016). Both animal and human studies have indicated that a portion of the dopamine neurons are activated by acute nociceptive stimuli (Brischoux et al. 2009; Budygin et al. 2012; Scott et al. 2006; Wood et al. 2007), that these dopaminergic responses are altered during chronic pain (see below), and that the mesostriatal system activity can influence the perception of pain. Thus, chronic pain tends to be exacerbated, and pain thresholds decreased by dopamine deficiency, such as that observed in humans with Parkinson’s disease or in animals after 6-hydroxydopamine lesions of the mesostriatal pathway (Saadé et al. 1997; Wood 2006; Zambito Marsala et al. 2011; Sung et al. 2018). Dopaminomimetic drugs, in contrast, exerted analgesic effects in tonic/chronic pain when administered into the dorsal or ventral striatum, and these effects were mediated by the dopamine D2, but not D1 receptor (Altier and Stewart 1993, 1998; Ansah et al. 2007; Magnusson and Fisher 2000).

A considerable body of evidence now suggests that chronic pain is accompanied by an attenuation of dopaminergic neurotransmission in the mesostriatal pathway, as indicated by reductions of basal extracellular striatal dopamine levels, dopaminergic cell firing, and dopaminergic responses to both noxious and rewarding stimuli (Ozaki et al. 2002; Wood et al. 2007; Taylor et al. 2014; Wu et al. 2014; Martikainen et al. 2015; Ren et al. 2016). Given the essentially antinociceptive actions of mesostriatal dopamine, these changes might be regarded as potential contributing factors to the development and maintenance of chronic pain. Moreover, human studies demonstrated morphological changes and altered functional connectivity of neurons within the striatum/NAc of chronic pain patients (Geha et al. 2008; Baliki et al. 2010; Baliki and Apkarian 2015). Altogether, these data suggest that functional and structural changes take place in both presynaptic and postsynaptic parts of the mesostriatal dopamine pathway during prolonged pain that are likely to have clinical relevance. However, the molecular underpinnings of these changes remain poorly understood.

In addition to its major dopaminergic input, the striatum is also characterized by high expression of most molecules involved in endogenous opioid signaling, critical players in the modulation of pain. Thus, it expresses two precursors of opioid peptides, proenkephalin (PENK) and prodynorphin (PDYN), as well as all three opioid receptors, mu, delta, and kappa (MOP, DOP, and KOP receptors, respectively) (Gerfen 1992; Mansour et al. 1995). PENK-derived peptides act predominantly on MOP and DOP receptors, and PDYN-derived peptides act predominantly on KOP receptors (Chavkin et al. 1982; cf. Yaksh and Wallace 2011). Whereas the classic sites of analgesic actions of opioids are located in the brainstem and the spinal cord (cf. Yaksh and Wallace 2011; Gilron et al. 2015), endogenous opioids are released into the striatum in response to nociceptive stimulation (Lapeyre et al. 2001; Zubieta et al. 2002), and activation of MOP and DOP receptors in the NAc results in analgesia (Dill and Costa 1977; Schmidt et al. 2002). Moreover, Zubieta et al. (2001) demonstrated in humans that both sensory and affective measures of pain were negatively correlated with MOP receptor stimulation in the NAc by endogenous opioids. In contrast to MOP and DOP receptors, striatal KOP receptors appear to be associated with antianalgesic effects (Schmidt et al. 2002). The above findings imply that endogenous opioid signaling within the striatum/NAc is strongly involved in pain processing.

Therefore, the molecular alterations underlying chronic pain could include changes in striatal opioid peptide or receptor expression or activity in addition to altered expression of molecules directly implicated in dopaminergic neurotransmission. This idea is supported by functional observations, e.g., in humans suffering from fibromyalgia or trigeminal neuralgia, decreased availability of MOP receptor was reported in the NAc (Harris et al. 2007; DosSantos et al. 2012). In animal models, some behavioral and neurochemical measures of dopamine reward system hypofunction associated with chronic pain could be reversed by inhibiting actions of striatal dynorphins on the KOP receptor (Suzuki et al. 1999; Narita et al. 2005). Excessive activation of the dynorphin–KOP system is known to produce depression-like emotional disturbances, such as anhedonia, which are also common to chronic pain conditions in humans (Knoll and Carlezon Jr 2010; Borsook et al. 2016).

The above evidence strongly suggests that neural processes mediated by both dopamine and opioid systems within the striatum/NAc play important roles in the regulation of descending systems for nociception control as well as in the affective response to the experience of chronic pain. Knowledge of the molecular alterations occurring in these systems during chronic pain, and neuropathic pain in particular, which is scarce at present, seems crucial to understanding their likely contribution to the development and maintenance of neuropathic pain conditions. Thus, we undertook the present study with the aim of providing a comprehensive profile of gene expression changes that occur in the NAc and dorsal striatum in a mouse model of neuropathic pain, considering a set of genes relevant to dopaminergic and opioidergic signaling within these forebrain regions. We demonstrate that strikingly many of these genes are upregulated in the NAc as a result of painful neuropathy.

## Materials and Methods

### Animals

Adult male Albino-Swiss CD-1 mice (20–25 g; Charles River, Sulzfeld, Germany) were used in this study. Animals were housed in groups of six in cages with sawdust bedding under a standard 12 h/12 h light/dark cycle (lights on at 06.00 a.m.); food and water were available ad libitum. The experiments were carried out according to the ethical standards and recommendations of the International Association for the Study of Pain (Zimmermann 1983), the European directive no. 2010/63/UE, and Polish regulations. The experimental protocol was approved by the Local Bioethics Committee (Krakow, Poland, permission number 1214/2015).

### Chronic Constriction Injury and Animal Treatment

Chronic constriction injury (CCI) to the sciatic nerve was performed under 1:1 10% ketamine/2% xylazine (*i.p.*) anesthesia using the procedure described by Bennett and Xie (1988). Briefly, an incision was made below the right hip bone, parallel to the sciatic nerve. The sciatic nerve was exposed, and three ligatures (4/0 silk) were tied loosely around the nerve distal to the sciatic notch with 1 mm spacing until a brief twitch in the respective hind limb was observed. According to our previous studies, such treatment results in neuropathic pain reflected by tactile and thermal hypersensitivity in the ipsilateral paw, which lasts for more than 3 weeks (Osikowicz et al. 2008; Mika et al. 2009). The study consisted of two experiments, one using the in situ hybridization (ISH) method and the other using quantitative reverse transcription–real-time PCR (qRT-PCR) and Western blot. The mice were sacrificed by cervical dislocation at 7 or 14 days after the operation for the ISH and qRT-PCR/Western blot analyses, respectively. Naive control groups were sacrificed simultaneously.

### Behavioral Tests

#### Von Frey’s Test

Mechanical tactile hypersensitivity in CCI mice was measured on the 7th and 14th day after CCI using a series of von Frey filaments (Stoelting, Wood Dale, IL, USA), ranging from 0.6 to 6 g (Mika et al., 2015). Animals were placed in plastic cages with a wire-mesh floor, allowing them to move freely. They were allowed to acclimate to this environment for approximately 5 to 15 min prior to testing. The von Frey filaments were applied in ascending order to the midplantar surface of each hind paw through the mesh floor. Each probe was applied to the foot until it started to bend. The ipsilateral and contralateral paws in CCI mice (or both hind paws in naive mice) were tested 2 to 3 times and a mean value was calculated. The time interval between consecutive applications of filaments was at least 5 s.

#### Cold Plate Test

Sensitivity to noxious thermal stimuli was assessed on the 7th and 14th day after CCI using a Cold/Hot Plate Analgesia Meter from Ugo Basile (Gemonio VA, Italy). The latency was defined as the amount of time it took for the hind paw to begin to shake after the mouse was placed on a cold plate (2 °C). In CCI mice, the injured paw reacted first in all cases. The ipsilateral paw reaction was noted first and then the contralateral paw response was awaited and noted. In naive mice, the reaction of any hind paw was noted. The cut-off latency for this test was 30 s (Mika et al., 2015).

### In Situ Hybridization

After sacrifice, the brains were removed and frozen on dry ice. They were then cut into 12-μm thick coronal sections on a cryostat microtome (CM 3050S Leica, Nussloch, Germany), the sections were thaw-mounted on gelatin-chrome alum-coated or Superfrost® Plus microscope slides (Gerhard Menzel, Braunschweig, Germany) and processed for ISH according to the method of Young et al. (1986). Briefly, the sections were fixed with 4% paraformaldehyde, washed in PBS and acetylated by incubation with 0.25% acetic anhydrite (in 0.1 M triethanolamine and 0.9% sodium chloride). The sections were then dehydrated using increasing concentrations of ethanol (70–100%), treated with chloroform for 5 min, and rehydrated with decreasing concentrations of ethanol.

The sections were hybridized for approximately 15 h at 37 °C with oligonucleotide probes for PENK, PDYN, or the dopamine D2 receptor mRNAs. The probes for the opioid propeptides were complementary to residues 388–435 and 862–909 of the rat PENK and PDYN gene transcripts, respectively (Yoshikawa et al. 1984; GenBank: K02807.1; Civelli et al. 1985; GenBank: M10088.1). Each probe contained one mismatch with respect to the corresponding mouse sequence (Zurawski et al. 1986; GenBank: NM_018863.2). The D2 receptor probe was complementary to residues 553–600 of the mouse D2 receptor mRNA (Short et al. 2006; NM_010077.1). All probes were labeled with ^35^S-dATP (Perkin Elmer, Waltham, MA, USA) by the 3′-tailing reaction using terminal transferase (MBI Fermentas, Vilnius, Lithuania).

After hybridization, the slices were washed three times for 20 min with 1 × SSC/50% formamide at 40 °C and twice for 50 min with 1 × SSC at room temperature. Then, the slices were dried and exposed to Fujifilm (Tokyo, Japan) phosphorimager imaging plates for 5 to 6 days. The hybridization signal was digitized using the Fujifilm BAS-5000 phosphorimager and Image Reader software.

### Image Analysis

The ISH signal was analyzed using the MCID Elite system (Imaging Research, St. Catharines, Ontario, Canada). Mean signal density, expressed in photostimulated luminescence units/mm^2^, was measured in selected brain regions in the Fujifilm BAS-5000 images. The regions included the anterior dorsal striatum (dStr), nucleus accumbens (NAc) core and shell (for PENK, PDYN, and the D2 receptor), substantia nigra pars compacta (SNc), and the ventral tegmental area (VTA) (for the D2 receptor only). The brain regions were delineated as previously (Ziolkowska et al. 2008; Martín-García et al. 2011) at coronal levels Bregma + 1.1 to 1.42 for the dStr and NAc, and Bregma − 2.92 to − 3.16 for the SNc and VTA (Paxinos and Franklin 2001). For each brain structure, data were collected from at least two sections per animal, separately for the brain side ipsilateral and contralateral to the injured nerve. The background signal, measured over brain section parts having the lowest optical density, was subtracted from the hybridization signal in the regions of interest.

### RNA Preparation and qRT-PCR

The brains were removed immediately after decapitation. They were transected along the sagittal fissure; the septum and the medial frontal cortex were pulled away to expose the interior of the lateral ventricle and the caudate putamen. A vertical cut was then made ventrally at the level of the anterior commissure and the tissue surrounding the commissure (nucleus accumbens) was pulled out using fine forceps. Tissue samples containing ipsilateral and contralateral nucleus accumbens were collected separately. They were placed in individual tubes with the tissue storage reagent RNAlater (Qiagen Inc., Valencia, CA, USA) and preserved at − 70 °C. The samples were thawed at room temperature and homogenized in TRIzol reagent (Invitrogen, Carlsbad, CA, USA). RNA isolation was performed in accordance with the manufacturer’s protocol. RNA concentration was measured using a NanoDrop ND-1000 Spectrometer (NanoDrop Technologies, Wilmington, DE, USA). Reverse transcription was performed on 1 μg of total RNA using Omniscript reverse transcriptase (Qiagen Inc.) at 37 °C for 60 min in the presence of RNase inhibitor (rRNAsin; Promega, Madison, WI, USA) and oligo (dT)12–18 primer (Invitrogen).

cDNA was diluted 1:10 with H_2_O. PCR was performed using Assay-On-Demand TaqMan probes according to the manufacturer’s protocol (Applied Biosystems, Foster City, CA, USA) and run on a real-time PCR iCycler (Bio-Rad, Hercules, CA, USA). The following TaqMan probes were used: Mm01545399_m1 for hypoxanthine guanine phosphoribosyl transferase 1 (HPRT1; *Hprt1* gene), Mm00457573_m1 for PDYN (*Pdyn* gene), Mm01212875_m1 for PENK (*Penk* gene), Mm01188089_m1 for the MOP receptor (*Oprm1* gene), Mm01230885_m1 for the KOP receptor (*Oprk1* gene), Mm01180757 for the DOP receptor (*Oprd1* gene), Mm01353211 for the dopamine D1 receptor (*Drd1* gene), and Mm00438545 for the dopamine D2 receptor (*Drd2* gene). The expression of HPRT1 (a housekeeping gene) was quantified to control for variation in cDNA amounts between samples. Threshold cycle values were calculated automatically by iCycler IQ 3.0 software with default parameters. The abundance of RNA was calculated as 2^−(threshold  cycle)^.

### Western Blot

Ipsilateral and contralateral nucleus accumbens were collected immediately after decapitation on day 14 after CCI (see the previous section for dissection details). The tissue samples were homogenized in RIPA buffer supplemented with a protease inhibitor cocktail. The homogenates were cleared by centrifugation (14,000×*g* for 30 min at 4 °C), and protein concentration was determined in the supernatant using the BCA Protein Assay Kit (Sigma-Aldrich, St. Louis, MO, USA). All samples (20 μg of protein from tissue) were heated in a loading buffer (4× Laemmli Buffer, Bio-Rad, Hercules, CA, USA) for 8 min at 98 °C. Next, the samples were resolved on 4–20% Criterion™ TGX™ precast polyacrylamide gels (Bio-Rad) and placed on Immune-Blot PVDF membranes (Bio-Rad) by the method of semidry transfer (30 min, 25 V). Membranes were blocked with 5% nonfat dry milk (Bio-Rad) in Tris-buffered saline with 0.1% Tween 20 (TBST) for 1 h at room temperature, washed with TBST, and incubated overnight at 4 °C with the following primary antibodies: rat anti-D1 (1:200, SantaCruz, sc-31478), rabbit anti-D2 (1:200, SantaCruz, CA, USA, sc-9113), and mouse anti-GAPDH (1:5000, Merck Millipore, Darmstadt, Germany, MAB374). Next, the membranes were incubated with horseradish peroxidase-conjugated anti-goat (1:1000, Vector, CA, USA, PI-9500), anti-rabbit (1:5000, Vector, PI-1000), or anti-mouse (1:5000, Vector, PI-2000) secondary antibodies for 1 h. We used the solutions from the SignalBoost™ Immunoreaction Enhancer Kit (Merck Millipore) in order to dilute the primary and secondary antibodies. The membranes underwent washing with TBST twice for 2 min each, and 3 times for 5 min each. In the final step, immune complexes were detected with the Clarity™ Western ECL Substrate (Bio-Rad) and visualized with a Fujifilm LAS-4000 FluorImager system. The relative levels of immunoreactive proteins were quantified densitometrically using the Fujifilm Multi Gauge software. After visualization, blots were washed again 2 times for 5 min each in TBST and reprobed with an antibody against GAPDH as an internal loading control. The levels of D1 and D2 receptors were normalized to internal references and presented as a ratio to the GAPDH signal.

### Statistical Analyses

The behavioral data are presented as the mean ± S.E.M. (*n* = 10 to 11). Intergroup differences were analyzed by two-way analysis of variance (ANOVA) considering time and brain side (ipsilateral vs. contralateral to the injury) as factors, which was followed by Bonferroni multiple comparison test. The qRT-PCR data are presented as the mean ± S.E.M. (*n* = 8 to 10). The results were analyzed using two-way ANOVAs considering treatment (naive vs. CCI) and brain side (ipsilateral vs. contralateral to the injury) as factors. The Western blot data are presented as the mean ± S.E.M. (*n* = 4 to 5). The results were evaluated using two-way ANOVAs to determine the effects of treatment and brain side. The ISH data are presented as the mean ± S.E.M. (*n* = 5 to 6). The results were analyzed by three-way ANOVAs in which the brain region was considered an additional factor. In all the analyses, *p* ≤ 0.05 was considered an indication of statistical significance.

## Results

### The Influence of CCI on Mechanical and Thermal Sensitivity Measured on Days 7 and 14 After Injury

**Fig. 1:**
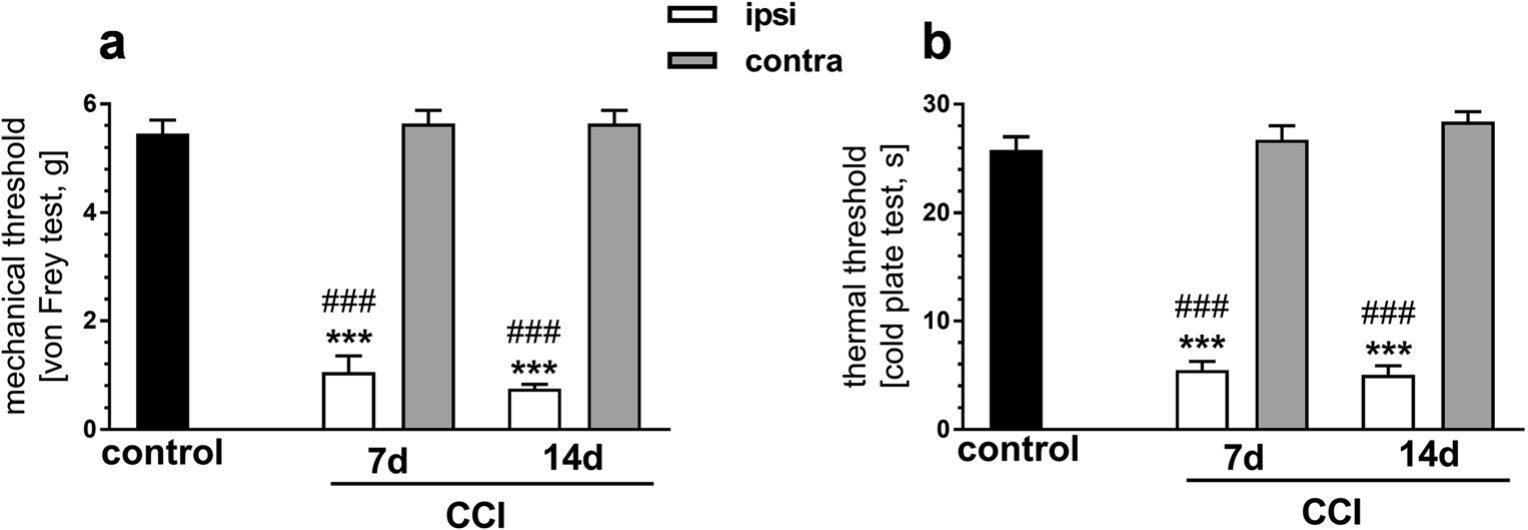
The effect of chronic constriction injury (CCI) on sensitivity to mechanical and thermal stimuli at 7 and 14 days after injury. **a** Sensitivity to mechanical stimuli as measured by the von Frey test. **b** Sensitivity to thermal stimuli as measured by the cold plate test. The data are presented as the mean (± S.E.M.) (*n* = 10 to 11). Intergroup differences were analyzed by two-way ANOVA followed by Bonferroni multiple comparison test. Triple asterisks indicate a significant difference compared with the control (naive) animals (*p* < 0.001). Triple number sign indicates a significant difference compared with the contralateral side on the same day after CCI (*p* < 0.001)

Pain thresholds in response to mechanical and thermal stimuli were measured by the von Frey and cold plate tests, respectively. CCI produced dramatic decreases in pain thresholds in the ipsilateral paw, as determined by both tests at 7 and 14 days after injury (Fig. 1). No changes in the response to both types of stimuli were observed in the contralateral paw at the time-points examined, as compared with the control group (Fig. 1).

### The Influence of CCI on Opioid Propeptide Gene Expression in the Striatum

**Fig. 2:**
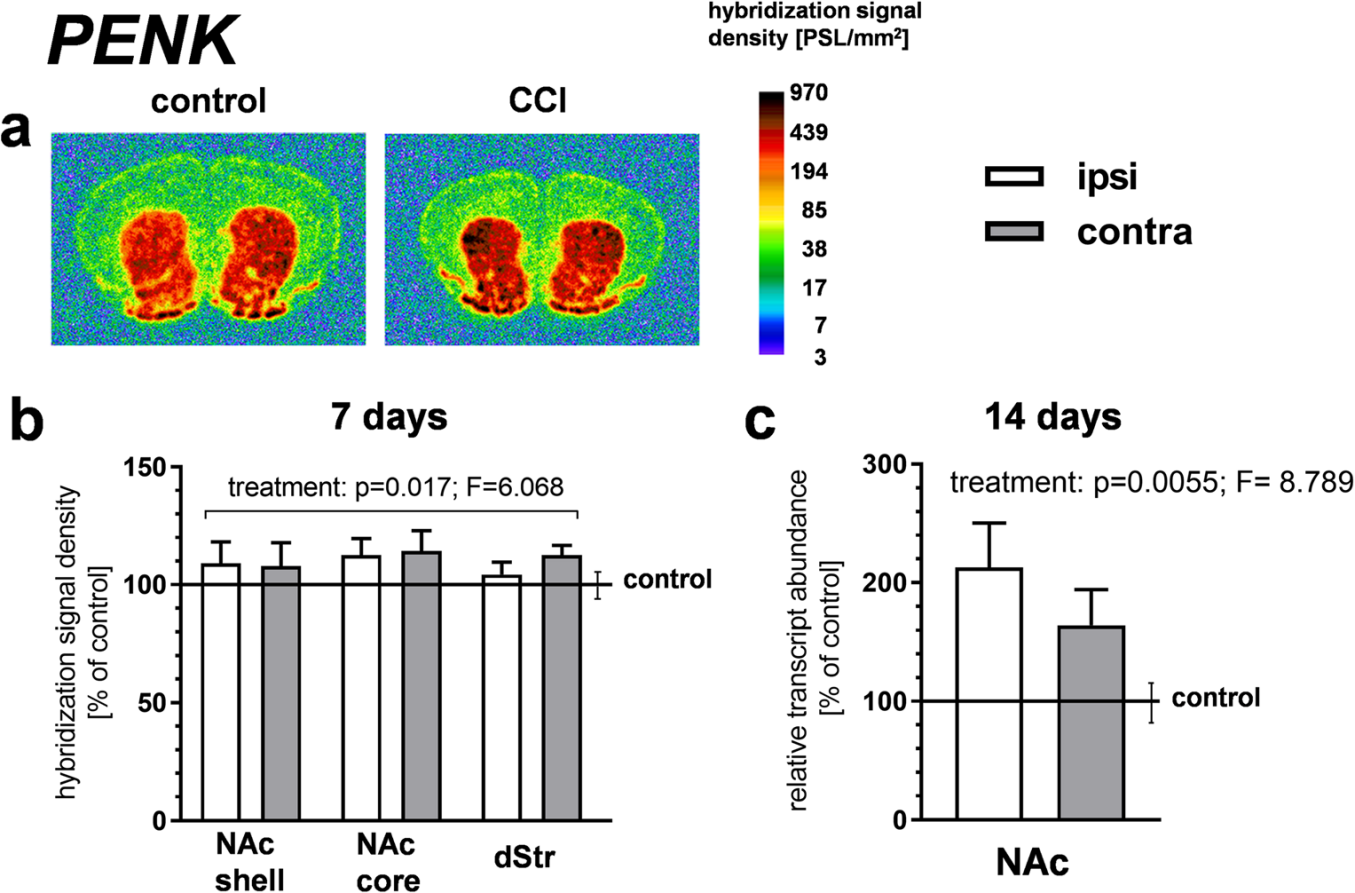
The effect of chronic constriction injury (CCI) on proenkephalin (PENK) gene expression in the striatum/nucleus accumbens. **a**, **b** An in situ hybridization analysis of PENK gene expression at 7 days after CCI. Panel **a** shows representative autoradiograms, where optical densities are color-coded according to the scale on the right (PSL, photostimulated luminescence units). The bars in panel **b** represent the mean (± S.E.M.) optical densities in the indicated brain regions of the CCI group (*n* = 6), ipsilateral and contralateral to the injured nerve. The data are expressed as a percentage of the corresponding values in the naive control group (*n* = 5). The control is indicated by the horizontal line at 100%, and the vertical error bar on the right-hand side of this line represents the mean S.E.M. in the control group. *p* and *F* values above the bars are the results of a three-way ANOVA (treatment × side × region of interest [ROI]). All other results of this ANOVA, not shown in the figure, were nonsignificant, except for the ROI factor (which reflected differences in the level of PENK gene expression between brain regions). **c** qRT-PCR measurements of PENK mRNA levels in the nucleus accumbens at 14 days after CCI. Data represent the mean (± S.E.M.) PENK transcript abundance standardized against *HPRT1* transcript and expressed as percentage of control (see description of panel **b**); *n* = 8 to 10. *p* and *F* values above the bars are the results of a two-way ANOVA (treatment × side). The side factor and treatment × side interaction were nonsignificant. The ANOVAs were performed on raw data. dStr, dorsal striatum; NAc, nucleus accumbens

Figure 2 a and b show the results of the ISH analysis of PENK gene expression in the striatal regions of interest (ROI) performed 7 days after CCI. A three-way ANOVA (treatment × side × ROI) revealed an influence of CCI on the expression of the PENK gene, showing significance of the treatment factor (*p* = 0.017; *F* = 6.068) in the absence of significant treatment × side or treatment × ROI interactions. These results indicate that CCI produced a bilateral increase in the PENK mRNA levels throughout the striatal regions tested, encompassing the dStr, NAc shell, and NAc core (Fig. 2a, b).

PENK gene expression was further studied using qRT-PCR (Fig. 2c). To assess the effects of longer-lasting pain, which could affect striatal gene transcription more profoundly, qRT-PCR measurements were performed 14 days after CCI; the measures of neuropathic pain, tactile, and thermal hypersensitivity remain at comparable levels between days 7 and 14 after CCI in mice (Fig. 1; Mika et al. 2009, 2015; Rojewska et al. 2018). Extracts of the whole NAc were used for this analysis. Two-way ANOVA (treatment × side) revealed a significant effect of the treatment factor (*p* = 0.0055; *F* = 8.789) on the expression of the PENK gene. Neither the side factor nor the treatment × side interaction proved significant, indicating a bilateral increase in the PENK transcript levels in the NAc (Fig. 2c).

**Fig. 3:**
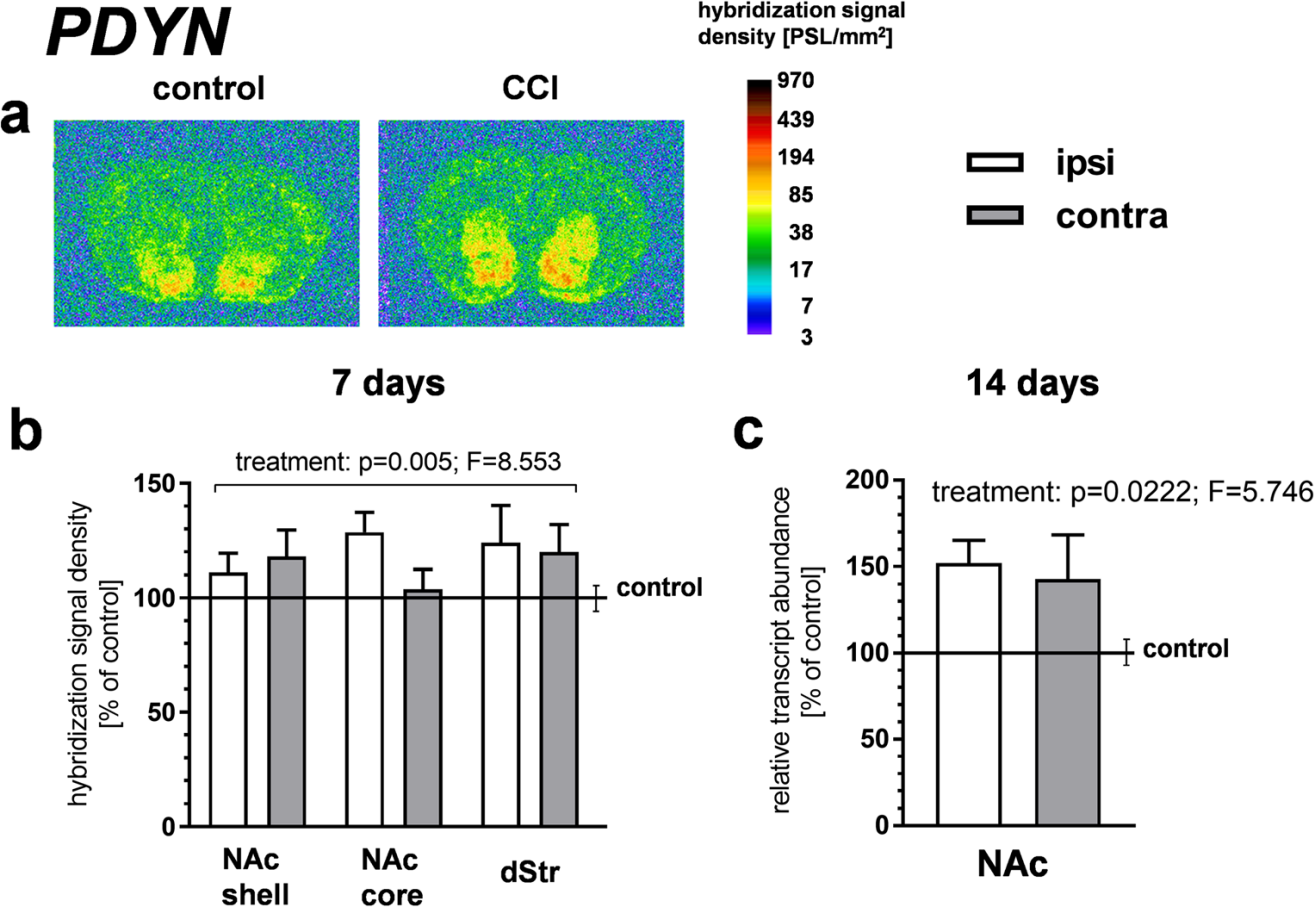
The effect of chronic constriction injury (CCI) on prodynorphin (PDYN) gene expression in the striatum/nucleus accumbens. **a**, **b** An in situ hybridization analysis of PDYN gene expression at 7 days after CCI. Panel **a** shows representative autoradiograms, where optical densities are color-coded according to the scale on the right (PSL, photostimulated luminescence units). The bars in panel **b** represent the mean (± S.E.M.) optical densities in the indicated brain regions of the CCI group (*n* = 6), ipsilateral and contralateral to the injured nerve. The data are expressed as a percentage of the corresponding values in the naive control group (*n* = 5). The control is indicated by the horizontal line at 100%, and the vertical error bar on the right-hand side of this line represents the mean S.E.M. in the control group. *p* and *F* values above the bars are the results of a three-way ANOVA (treatment × side × region of interest [ROI]). All other results of this ANOVA, not shown in the figure, were nonsignificant, except for the ROI factor (which reflected differences in the level of PDYN gene expression between brain regions). **c** qRT-PCR measurements of PDYN mRNA levels in the nucleus accumbens at 14 days after CCI. Data represent the mean (± S.E.M.) PDYN transcript abundance standardized against *HPRT1* transcript and expressed as percentage of control (see description of panel **b**); *n* = 8 to 10. *p* and *F* values above the bars are the results of a two-way ANOVA (treatment × side). The side factor and treatment × side interaction were nonsignificant. The ANOVAs were performed on raw data. dStr, dorsal striatum; NAc, nucleus accumbens

The PDYN gene was regulated by CCI in a similar fashion as PENK (Fig. 3). Thus, CCI produced a bilateral upregulation of the PDYN gene in striatal regions at 7 days as demonstrated by a three-way ANOVA of the ISH results (significant treatment factor, *p* = 0.005, *F* = 8.553; treatment × side and treatment × ROI interactions nonsignificant; Fig. 3a, b). Elevated levels of PDYN mRNA were also observed by qRT-PCR in the NAc at 14 days after CCI (Fig. 3c).

### The Influence of CCI on Opioid Receptor Gene Expression in the Striatum

**Fig. 4:**
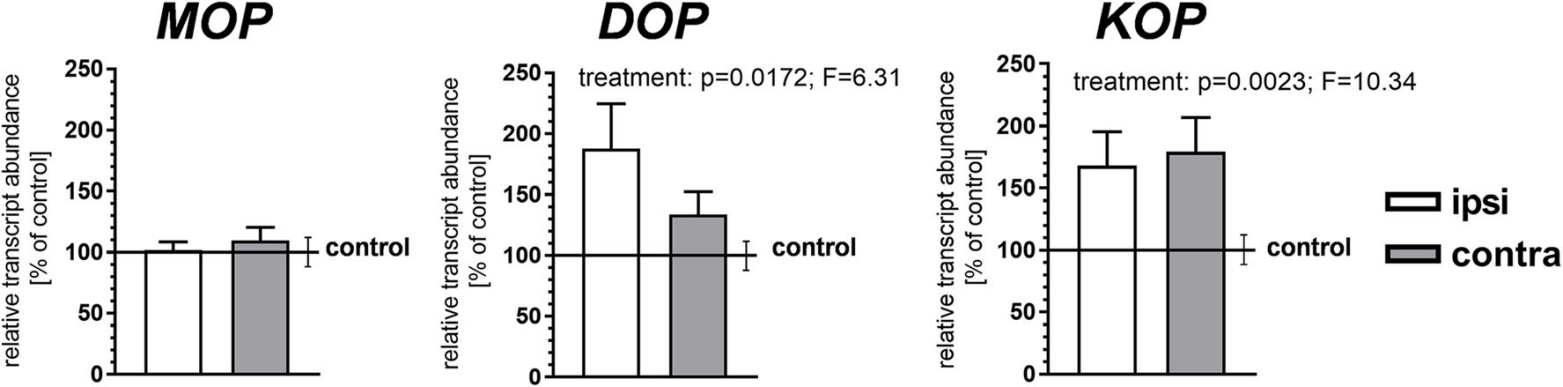
The effect of chronic constriction injury (CCI) on MOP, DOP, and KOP opioid receptor gene expression in the nucleus accumbens. The bars show the results of qRT-PCR measurements of MOP, DOP, and KOP mRNA levels in the nucleus accumbens at 14 days after CCI. Data represent the mean (± S.E.M.) transcript abundance standardized against *HPRT1* transcript and expressed as percentage of control. The control is indicated by the horizontal line at 100%, and the vertical error bars on the right-hand side of these lines represent the mean S.E.M. in the control group; *n* = 8 to 10. *p* and *F* values above the bars are the results of a two-way ANOVA (treatment × side). The side factor and treatment × side interactions were nonsignificant. ANOVAs were performed on raw data

Due to the low abundance of the opioid receptors transcripts in the striatum and low sensitivity of the ISH method applied, expression of these receptors was assessed only by qRT-PCR. CCI produced bilateral upregulation of the DOP and KOP receptor transcripts in the NAc at 14 days (*p* = 0.0172, *F* = 6.31 and *p* = 0.0023, *F* = 10.34 for the treatment factor, respectively), whereas the level of the MOP receptor transcript remained unchanged (Fig. 4).

### The Influence of CCI on Dopamine Receptor mRNA and Protein Levels in the Mesostriatal System

**Fig. 5:**
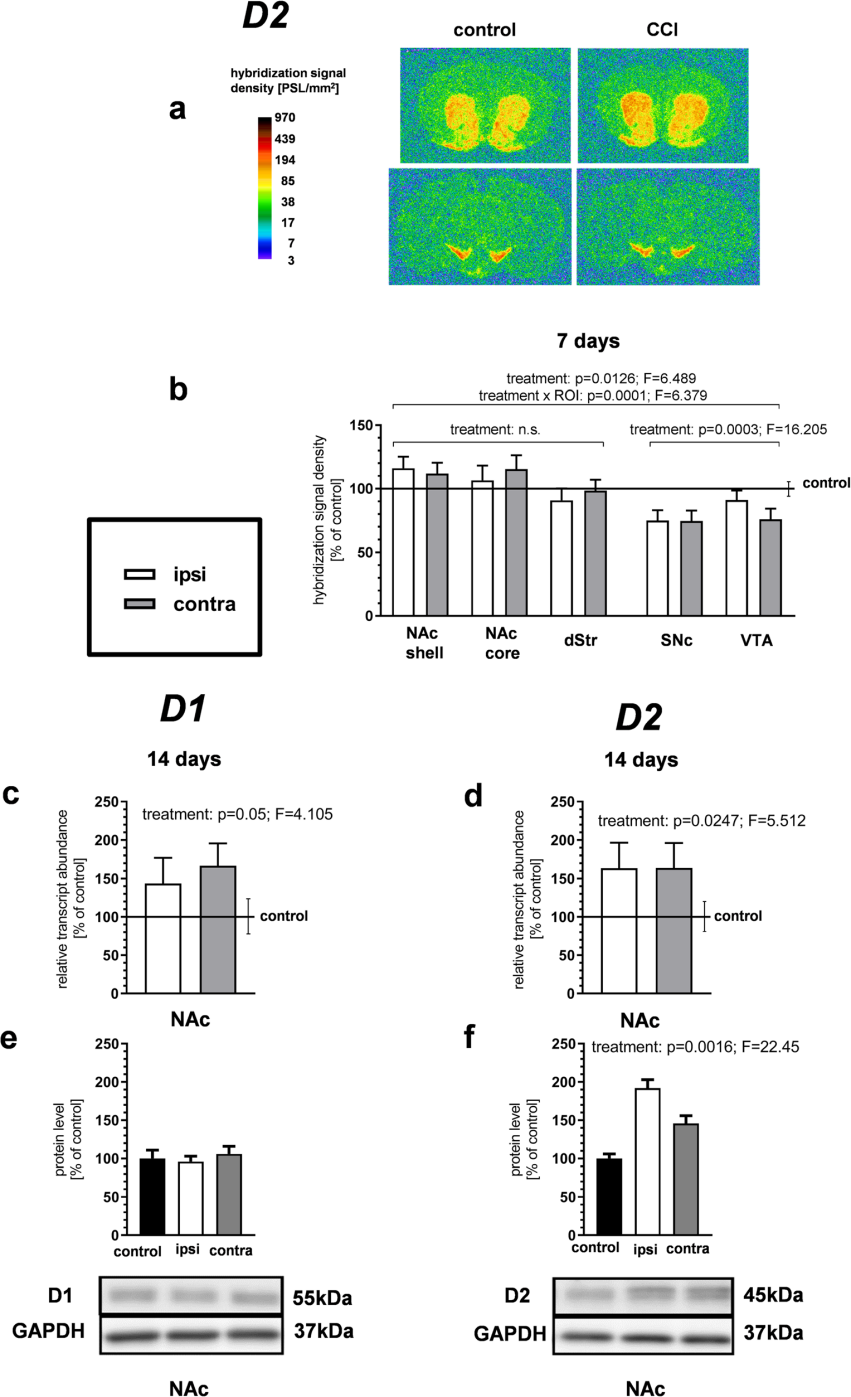
The effect of chronic constriction injury (CCI) on the dopamine D1 and D2 receptor mRNA and protein levels in the mesostriatal system. **a**, **b** An in situ hybridization analysis of D2 receptor gene expression at 7 days after CCI. Panel **a** shows representative autoradiograms, where optical densities are color-coded according to the scale on the left (PSL, photostimulated luminescence units). The bars in panel **b** represent the mean (± S.E.M.) optical densities in the indicated brain regions of the CCI group (*n* = 6), ipsilateral and contralateral to the injured nerve. The data are expressed as a percentage of the corresponding values in the naive control group (*n* = 5). The control is indicated by the horizontal line at 100%, and the vertical error bar on the right-hand side of this line represents the mean S.E.M. in the control group. *p* and *F* values above the bars are the results of three-way ANOVAs (treatment × side × region of interest [ROI]). All other results of these ANOVAs, not shown in the figure, were nonsignificant, except for the ROI factor (which reflected differences in the level of the D2 receptor gene expression between brain regions). **c**, **d** qRT-PCR measurements of the D1 and D2 receptor mRNA levels in the nucleus accumbens at 14 days after CCI. Data represent the mean (± S.E.M.) transcript abundance standardized against *HPRT1* transcript and expressed as percentage of control (see description of panel **b**); *n* = 8 to 10. *p* and *F* values above the bars are the results of two-way ANOVAs (treatment × side). The side factor and treatment × side interaction were nonsignificant. **e**, **f** Western blot measurements of the D1 and D2 receptor protein levels in the nucleus accumbens at 14 days after CCI. Data represent the mean (± S.E.M.) expressed as percentage of control; *n* = 4 to 5. p and *F* values above the bars are the results of two-way ANOVAs (treatment × side). The side factor and treatment × side interaction were nonsignificant in both cases. For the D1 receptor, the treatment factor was also nonsignificant. All ANOVAs were performed on raw data. dStr, dorsal striatum; NAc, nucleus accumbens; SNc, substantia nigra pars compacta; VTA, ventral tegmental area

Considering the high expression of the dopamine D2 receptor in mesencephalic dopamine neurons, the levels of D2 receptor mRNA were assessed by ISH in the SNc and VTA in addition to the striatal regions (Fig. 5a, b). Three-way ANOVA (treatment × side × ROI) revealed the significance of both treatment factor and treatment × ROI interaction (but not of the treatment × side interaction), thus demonstrating differential regional regulation of the D2 receptor mRNA levels at 7 days after CCI (Fig. 5b). To make the analysis comparable with that performed for the other genes, we then carried out ANOVAs separately for the striatal regions (NAc shell, core and dStr, as in the case of PENK and PDYN) and mesencephalic regions (SNc and VTA). The effect of the treatment was not significant by a three-way ANOVA considering only the striatal regions, even though a tendency toward an increase in the D2 receptor mRNA levels was observed in the NAc. In contrast, a significant decrease in D2 receptor expression was detected in the mesencephalic regions (*p* = 0.0003, *F* = 16.205 for the treatment factor; Fig. 5b). The lack of a significant treatment ×side interaction in this analysis suggested that the effect was bilateral.

A two-way ANOVA of qPCR measurements at 14 days after CCI demonstrated a bilateral increase in the D2 receptor mRNA levels in the NAc (*p* = 0.0247, *F* = 5.512 for the treatment factor and nonsignificant treatment × side interaction; Fig. 5d). This was accompanied by an increase in the protein level of the D2 receptor, as determined by Western blot at the same time-point (*p* = 0.0016, *F* = 22.45 for the treatment factor and nonsignificant treatment ×side interaction; Fig. 5f).

An increase in mRNA levels was also observed for the D1 receptor in the NAc (*p* = 0.05, *F* = 4.105 for the treatment factor; Fig. 5c). However, the levels of the corresponding protein remained unchanged in the NAc at 14 days (Fig. 5e).

## Discussion

Our results demonstrated that chronic constriction sciatic nerve injury produced increases in gene expression of the dopamine and opioid receptors, as well as opioid propeptides, in the NAc. They also suggested that at least some of these genes were upregulated in the dStr as well. In addition, pain-related transcriptional alterations seemed to occur not only in the target regions of the dopaminergic mesostriatal pathways but also in the dopaminergic neurons themselves, as exemplified by downregulated levels of the D2 receptor mRNA in the VTA and SNc of mice subjected to CCI.

Decreased dopamine signaling in the mesolimbic system has been reported in neuropathic pain models, including decreased dopaminergic cell firing, extracellular dopamine levels in the NAc, and reactivity to rewarding stimuli (Ozaki et al. 2002; Taylor et al. 2014; Wu et al. 2014; Ren et al. 2016). The function of dopamine neurons is regulated by dopamine via inhibitory somatodendritic and axonal D2 autoreceptors, whose stimulation limits dopaminergic cell firing and neurotransmitter release (cf. Ford 2014). Thus, the downregulation of the D2 receptor gene in mesencephalic dopaminergic cell body regions observed in our study can be regarded as a potential compensatory change, provided that it translates into a corresponding drop in functional protein levels. It could act to partially restore dopamine system function by limiting the D2 receptor-mediated autoinhibitory control of dopamine over its neurons. Interestingly, similar changes in the D2 receptor mRNA levels were reported in the VTA and SNc after chronic treatment of rats with other stressful procedures, which did not include painful stimuli (Dziedzicka-Wasylewska et al. 1997).

Upregulation of postsynaptic D1 and D2 receptors in the NAc observed in our study after CCI could have a similar net effect of compensating for the deficient neurotransmission by the presynaptic dopaminergic neurons. Nevertheless, our measurements of the receptor protein levels in the NAc indicate that only the D2 (but not D1) receptor may actually play such a role. This is because, in contrast to the D2 receptor protein abundance, which increased in the NAc after CCI, the D1 receptor protein level remained unchanged (despite upregulation of the corresponding mRNA). Other studies regarding expression of the accumbal dopamine receptors in rodent models of neuropathy yielded inconsistent results demonstrating upregulation, downregulation, or lack of change in the D1 and D2 receptor mRNA and/or protein expression (Austin et al. 2010; Chang et al. 2014; Sagheddu et al. 2015). Importantly, the work by Chang et al. (2014) suggested that regulation of these receptors expression may depend on the duration of pain, which may explain major differences in results obtained using various experimental paradigms.

Similar to other parts of the striatum, the NAc is composed mainly of GABAergic projection medium spiny neurons (MSN). They comprise two major cell classes, one of which (D1-MSN) is characterized by the expression of the dopamine D1 receptor, substance P, and the opioid propeptide PDYN, as well as by a predominant projection to the mesencephalon (the so-called direct striatofugal pathway). The other MSN type (D2-MSN) is characterized by the expression of the D2 receptor and the opioid propeptide PENK, and by a predominant projection to the pallidum (the so-called indirect pathway) (Gerfen et al. 1990; Gerfen 1992; Sesack and Grace 2010). While the expression of these receptors and neuropeptides is strongly segregated in the dStr, the NAc contains a larger proportion of neurons of mixed molecular phenotypes and projection targets (Curran and Watson 1995; Zhou et al. 2003; Perreault et al. 2010; Kupchik et al. 2015).

Our experiment demonstrated that markers of both striatofugal pathways (D1 receptor and PDYN, as well as D2 receptor and PENK) are upregulated at the transcriptional level in the CCI model of neuropathic pain in the NAc. Due to differences in D1 vs. D2 receptor coupling, dopamine exerts opposite effects on the two striatal MSN populations: it stimulates D1-MSN and inhibits D2-MSN (Gerfen and Surmeier 2011). The neuropathic pain-related decrease in dopaminergic tone could lead to disinhibition of the D2-MSN via the D2 receptor and contribute to the observed increases in the D2 receptor and PENK mRNA levels. In fact, these two genes are known to be upregulated in the dStr by dopamine deficiency or by blockade of the D2 receptor (Young et al. 1986; Gerfen et al. 1990; Reimer et al. 1992). On the other hand, some markers of the D1-MSN, including the PDYN gene, are positively regulated by dopamine via the D1 receptor (Berke et al. 1998; Gerfen et al. 1990; Reimer et al. 1992). Thus, their upregulation in the neuropathic pain model used in our experiments cannot be attributed to the decreased dopaminergic tone.

Upregulation of the dopamine receptors and opioid propeptides mRNAs in both subpopulations of MSN could result from alterations in glutamatergic neurotransmission. The NAc is densely innervated by glutamatergic afferents that impinge on all MSNs (Ghasemzadeh et al. 1996; Sesack and Grace 2010). In chronic neuropathic and inflammatory pain models, increased synaptic levels of the specific GluA1-rich AMPA glutamate receptors, which are permeable to calcium ions, were reported in the NAc (Goffer et al. 2013; Su et al. 2015). It seems plausible that increased calcium influx into the MSN via these receptors might promote Ca^2+^-dependent transcriptional mechanisms, including activation of the transcription factor CREB (cAMP response element binding protein) (Ginty 1997; Perkinton et al. 1999), and result in upregulation of its target genes, such as PDYN and PENK (Konradi et al. 1993, 1995; Cole et al. 1995).

The increased levels of transcripts characteristic of both the direct and indirect striatofugal pathway suggest that the net excitatory input to D1-MSN and D2-MSN is strengthened after CCI and thus indicates that both efferent pathways originating in the striatum might be overactive in the neuropathic pain model. Whereas the role of the direct pathway and the accumbal D1 receptor in pain perception is not clear, ample evidence shows that activity of the indirect pathway D2-MSN in the NAc is important for chronic pain sensation. Namely, targeting this neuronal population by a chemogenetic method or selective D2 receptor ligands demonstrated that stimulation of the indirect pathway exerted proalgesic effects, whereas its inhibition reduced mechanical and thermal hypersensitivity in neuropathic pain and formalin models (Altier and Stewart 1993, 1998; Magnusson and Fisher 2000; Ansah et al. 2007; Cobacho et al. 2014; Ren et al. 2016). Thus, our results suggest that peripheral neuropathy affects the accumbal indirect pathway D2-MSN activity in such a manner that it can enhance the perception of pain.

The opioid propeptide genes PDYN and PENK were upregulated in the NAc of CCI-subjected animals in our study. Moreover, the expression of the KOP and DOP receptors, which are the preferential binding targets for PDYN- and PENK-derived peptides, respectively, was also increased at the transcriptional level. These observations suggest that endogenous opioid signaling within the NAc might be strengthened in neuropathic pain.

This is in contrast to our previous observations of decreased expression and activity (GTPγS binding) of opioid receptors in the spinal cord and thalamus of mice subjected to the same CCI procedure, accompanied by upregulation of the spinal PENK and PDYN genes (Rojewska et al. 2018). Studies in different experimental settings also yielded some contrasting results regarding regulation of opioid receptors in animal models of neuropathic pain. For example, in rat models, decreased mRNA levels of the DOP and KOP receptor were reported in the NAc at delayed time-points (1 month) but not at 1 week after nerve injury (Llorca-Torralba et al. 2020 and Chang et al. 2014, respectively). However, these gene expression changes were not paralleled by analogous changes in agonist-stimulated receptor activity (GTPγS binding), although the latter was altered in a manner apparently independent on the corresponding receptor transcript levels (Llorca-Torralba et al. 2020). On the other hand, consistent with our results, elevation of both KOP receptor mRNA and agonist-stimulated activity was demonstrated in the mouse at 14 days after peripheral nerve injury (Liu et al. 2019). Discrepancies in the existing data indicate the necessity of further research into the regulation of the striatal opioid receptors by chronic pain.

Interestingly, the changes demonstrated by our group in the spinal cord and thalamus, which contain components of the somatosensory pathways, were unilateral and limited to the side conveying the ascending nociceptive information from the injured hindpaw nerve (Rojewska et al. 2018). In contrast, all changes detected in the present study in the striatum/NAc were bilateral, consistent with the concept of the NAc (and its local opioids) involvement in a more generalized control over pain thresholds and the affective aspect of pain (Baliki and Apkarian 2015; Borsook et al. 2016).

The best characterized effect of striatal dynorphins is the inhibition of dopamine release via stimulation of the KOP receptor located on dopaminergic axon terminals (Di Chiara and Imperato 1988; Spanagel et al. 1992). This mechanism has been considered responsible for the fact that KOP receptor agonists produce aversion and anhedonia (Bals-Kubik et al. 1989; Knoll and Carlezon Jr 2010; Tejeda et al. 2012). These affective disturbances are common to chronic pain conditions, and deficits of natural and drug reward are observed in animal models thereof (Suzuki et al. 1999; Narita et al. 2005; Borsook et al. 2016). Moreover, pain-related reward deficits could be reversed by intra-NAc infusions of a KOP receptor antagonist or an antibody against dynorphin (Suzuki et al. 1999; Narita et al. 2005). Therefore, it has been posited that protracted pain might be associated with an increase in endogenous dynorphin/KOP receptor signaling in the NAc (Niikura et al. 2010; Borsook et al. 2016; Massaly et al. 2016). Our results provide a potential molecular mechanism for this proposal by demonstrating increased expression of the accumbal PDYN gene in the CCI model of neuropathic pain, which presumably results in elevated levels of available striatal dynorphins (Massaly et al. 2019). Even though some other studies did not reveal such changes (Chang et al., 2014; Leitl et al. 2014; Palmisano et al. 2018), our observation is in line with the very recent papers by Liu et al. (2019) and Massaly et al. (2019). The latter two studies also provide functional evidence for the role of the increased mesolimbic dynorphin/KOP receptor signaling in mediating the tonic aversive component of neuropathic and inflammatory pain. Accordingly, our previous experiments in inbred mouse strains have demonstrated that high levels of PDYN mRNA in the dStr/NAc may be associated with low sensitivity to drug reward (Gieryk et al. 2010).

The proposed role of striatal dynorphins in chronic pain consists of attenuation of dopamine release via the presynaptic KOP receptor (Narita et al. 2005; Massaly et al. 2016). However, our experiment suggests that other mechanisms of potentiated dynorphin/KOP receptor signaling could also be involved because, in agreement with Liu et al. (2019), it shows upregulated KOP receptor gene expression in the postsynaptic (rather than the dopaminergic presynaptic) neurons of the NAc. According to the recent study by Tejeda et al. (2017), stimulation of the KOP receptor located on MSN collaterals in the NAc, in addition to those expressed by glutamatergic afferents from the basolateral amygdala, led to a selective disinhibition of the accumbal D2-MSN. Since the activity of this neuronal population is linked to thermal and mechanical hypersensitivity in persistent pain models (see above), it is tempting to speculate that the CCI-produced PDYN and KOP receptor gene expression changes may contribute to the increased pain sensitivity. However, the KOP receptor is expressed by several types of neurons in the NAc, including both MSN populations and some interneurons (Oude Ophuis et al. 2014; Tejeda et al. 2017). Knowledge of the cell types that overexpress the KOP receptor in chronic pain, not available at present, would be crucial for predicting the effects of its upregulation on specific neuronal circuits activity.

In contrast to the effects of dynorphins in the Str/NAc, increased expression of PENK and its derived peptides acting on the MOP and DOP receptors can function as a negative feedback mechanism to limit the excessive stimulation of the striatopallidal pathway D2-MSN due to chronic pain-related deficiency in dopaminergic signaling. Such a feedback mechanism may operate on several levels, involving enkephalins’ actions within the Str/NAc (i) on the corticostriatal glutamatergic terminals, whereby enkephalins reduce glutamate release and the resulting postsynaptic excitation of the striatal MSN (Jiang and North 1992; Miura et al. 2007), as well as (ii) on D2-MSN dendrites and somata containing MOP and DOP receptors (Oude Ophuis et al. 2014; Banghart et al. 2015), whereby enkephalins can prevent, e.g., transcriptional activation of these neurons resulting from D2 receptor blockade (Steiner and Gerfen 1999). Enkephalins can also hamper neurotransmission by the striatopallidal MSN by acting on their axon terminals in the ventral pallidum. These terminals contain MOP receptors (Olive et al. 1997), whose stimulation by exogenous or endogenous agonists seems to reduce the release of GABA, i.e., the main neurotransmitter in the striatopallidal pathway (Kupchik et al. 2014). Considering that overactivity of this pathway exacerbates chronic pain, enhanced enkephalin signaling via the MOP and DOP receptors (resulting from increased expression of both PENK and DOP, but not MOP, receptor genes) is expected to produce some analgesia in the CCI model used in our experiments. Such a role of endogenous enkephalins is supported by the observation of analgesic actions of MOP and DOP receptor agonists administered locally into either the NAc or the pallidum (Dill and Costa 1977; Schmidt et al. 2002; Anagnostakis et al. 1992). Nevertheless, it is clear that pronociceptive mechanisms recruited concomittantly in the NAc, as well as in other parts of the nervous system during neuropathy, prevail over the antinociceptive actions likely exerted by enkephalins.

Altogether, we posit that the demonstrated changes in gene expression of the opioid propeptides and their respective receptors, i.e., PENK and DOP receptor vs. PDYN and KOP receptor, in neuropathy induced by sciatic nerve injury, may exert opposite influence on pain perception. Increased activity of the PENK/DOP (and MOP) receptor system within the NAc and/or pallidum is likely to suppress pain to some extent but it is apparently dominated by the enhanced PDYN/KOP receptor signaling in the NAc, which probably exacerbates chronic pain and promotes the associated dysphoric emotional disturbances. Thus, we suggest that a disturbed balance between endogenous opioid systems in favor of the pronociceptive one may contribute to the development of mechanical and thermal hypersensitivity in the CCI model of neuropathy.
